# Nocturnal Emissions; a Query

**Published:** 1885-08-20

**Authors:** 


### EDITORIALS AND MISCELLANEOUS.

### NOCTURNAL EMISSIONS.

A QUERY.—Dr. E. A. Hinton, of Alabama, writes for an opinion as to the following prescription as a remedy for nocturnal emissions :

℞. Dilute phosphoric acid.......................................................ʒ i.
Take at each meal, and
Tinc. Gelsemium ...........................................................gtts. xx
between each meal ; and give before each meal
Tinc. nux vomica...........................................................gtts. x.
He adds, that " the irritability of the bladder will be relieved by
Carbonate of Lithia.........................................................grs. v
in water, three times a day."

He asks, " Is it an inevitable consequence that, using the above treatment without the Lithia, the bladder will be irritated ? "

[We reply, that the Carbonate of Lithia will relieve only the irritability which results from excess of uric acid deposits, and yet how it diminishes, and even checks such deposits, is not understood ; nor can it be satisfactorily explained how that phosphoric acid seems to relieve that form of irritability resulting from an excess of phosphates in the urine. The nux is the essential agent in the above prescription, and given alone, or with some preparation of iron, quinia or like tonic, will better meet the indications in a majority of such cases. The gelsemium, though useful in allaying the irritability of the bladder, where it exists, antagonizes the nux, and should be used only in that class of cases where there is a good degree of vigor, and not in feeble or anœmic cases, and then only to allay undue excitement, and produce rest at night.—EDITOR.]

